# The study protocol of a blinded randomised-controlled cross-over trial of lavender oil as a treatment of behavioural symptoms in dementia

**DOI:** 10.1186/1471-2318-10-49

**Published:** 2010-07-22

**Authors:** Eva S van der Ploeg, Barbara Eppingstall, Daniel W O'Connor

**Affiliations:** 1 Aged Mental Health Research Unit, Monash University, Kingston Centre, Warrigal Road, Cheltenham, Melbourne, VIC 3192, Australia

## Abstract

**Background:**

The agitated behaviours that accompany dementia (e.g. pacing, aggression, calling out) are stressful to both nursing home residents and their carers and are difficult to treat. Increasingly more attention is being paid to alternative interventions that are associated with fewer risks than pharmacology. *Lavandula angustifolia *(lavender) has been thought, for centuries, to have soothing properties, but the existing evidence is limited and shows mixed results. The aim of the current study is to test the effectiveness of topically applied pure lavender oil in reducing actual counts of challenging behaviours in nursing home residents.

**Methods/Design:**

We will use a blinded repeated measures design with random cross-over between lavender oil and placebo oil. Persons with moderate to severe dementia and associated behavioural problems living in aged care facilities will be included in the study. Consented, willing participants will be assigned in random order to lavender or placebo blocks for one week then switched to the other condition for the following week. In each week the oils will be applied on three days with at least a two-day wash out period between conditions. Trained observers will note presence of target behaviours and predominant type of affect displayed during the 30 minutes before and the 60 minutes after application of the oil. Nursing staff will apply 1 ml of 30% high strength essential lavender oil to reduce the risk of missing a true effect through under-dosing. The placebo will comprise of jojoba oil only. The oils will be identical in appearance and texture, but can easily be identified by smell. For blinding purposes, all staff involved in applying the oil or observing the resident will apply a masking cream containing a mixture of lavender and other essential oils to their upper lip. In addition, nursing staff will wear a nose clip during the few minutes it takes to massage the oil to the resident's forearms.

**Discussion:**

If our results show that the use of lavender oil is effective in reducing challenging behaviours in individuals with dementia, it will potentially provide a safer intervention rather than reliance on pharmacology alone. The study's findings will translate easily to other countries and cultures.

**Trial Registration:**

Australian New Zealand Clinical Trials Registry - ACTRN 12609000569202

## Background

The challenging behaviours that accompany dementia (e.g. pacing, aggression and calling out) can be stressful for nursing home residents as well as their carers and are difficult to treat. Such behaviours can be a consequence of multiple causes. Some are due to pain, depression or psychosis and require treatment with analgesia or psychotropic medications. In other cases, atypical antipsychotic medications are also the preferred treatment [1] but have shown modest efficacy and can have adverse effects such as increased confusion and falls [2,3]. Therefore, novel treatments should be explored. An example is *Lavandula angustifolia *(lavender) which has been thought, for centuries, to have soothing properties that may affect the anxiety and agitation that underlie the behavioural symptoms associated with dementia.

Lavender is absorbed through skin and respiration and acts on neurotransmitter systems. Linalool (an essential ingredient of lavender) was shown by Elisabetsky *et al*. [4] to inhibit glutamate binding in rat cortex in a dose-dependent fashion, consistent with its putative sedative and anticonvulsant properties. When mice were exposed to constant airborne lavender fragrance, their plasma levels of linalool and linalyl acetate, the primary constituents of lavender, peaked at about 60 minutes [5]. Furthermore, rodents' motor activity fell to 20% of baseline levels within 30 minutes of exposure to lavender fragrance and within 60 minutes, mice showed typically "sedated" behaviour, crouching motionless in a corner of their cage. When they were injected later with caffeine, a rise in motor activity was blocked by simultaneous exposure to lavender. Lemon balm and other essential oils had much less effect [6].

In humans, plasma levels of linalool and linalyl acetate rose quickly when 2% lavender oil was massaged into abdominal skin over a 10 minute period [7]. Diego *et al*. [8] exposed 40 university staff and students to lavender and rosemary oils for periods of three minutes while monitoring their EEG, mood state, attention and mathematical accuracy. Lavender, but not rosemary, was associated with improved mood and accuracy and with increased frontal alpha power, consistent with drowsiness. In a single case evaluation, Brooker *et al*. [9] observed variable effects after treating four psychogeriatric patients for 10-minute periods on ten occasions each with lavender oil by vapour, massage with a neutral oil and vaporised lavender oil combined with massage. When compared with 'no treatment' control sessions, only one participant benefited to a statistically significant degree and two became more agitated. Two other case series suggested that lavender promotes sleep in elderly people with dementia [10,11].

Three trials found no significant changes in behavioural symptoms following exposure to lavender [12-14], however two trials showed positive effects. Holmes *et al*. [15] sprayed the communal area of a dementia ward with either 2% Lavender oil or water for two hours daily on alternating days. All 15 participants had severe dementia and daily agitation. An observer wearing a nose clip rated behaviours using the Pittsburgh Agitation Scale in the final hour of 10 sessions. Median behaviour scores were 20% lower while exposed to lavender compared to water (p = 0.016). More recently, Lin *et al*. [16] administered lavender or sunflower oils by vapour for an hour each night while nursing home residents were asleep. All 70 participants had marked dementia with clinically significant agitation. In a repeated measures study with randomised cross-over, both oils were administered for three week periods, with a two-week washout between. Agitation scores fell by 7% with lavender compared with less than 1% with sunflower oil (p < 0.001).

A recent review concluded that while aromatherapy, amongst other non-pharmacological treatments, was identified as a potential treatment of behavioural problems in dementia, studies were often weakened by small sample sizes, lack of controls and imprecise measures [17]. Furthermore, previous studies generally failed to specify source, purity, concentration and reliability of delivery.

Therefore, the aim of the proposed study is to test the effectiveness of topically applied 30% lavender oil of proven purity as a treatment of behavioural symptoms in dementia. To this end, we will conduct a randomised-controlled trial (RCT) by way of a cross over design with an appropriate control condition and blinding. We hypothesise that lavender oil will lead to a greater reduction in the frequency of behavioural symptoms of dementia than a placebo oil.

## Methods/Design

### Study design

We will test our hypothesis using a blinded, repeated measures design with random cross-over between conditions. Repeated measures RCTs minimise intra and inter-individual differences since all participants are subject to both conditions and behaviours vary greatly in frequency within and between people with dementia from hour to hour and day to day [17].

### Ethical considerations

The protocol has been approved by the ethics committees of Monash University and all the respective health organisations to which the participating aged care facilities (ACFs) are affiliated (Southern Health, Peninsula Health, St Vincent's Health and Alfred Health).

It is most unlikely that participants can provide informed consent. In Victoria, the "person responsible" (usually a spouse or child) consents to the participation of non-competent persons with dementia in non-invasive studies. While the person responsible provides written, informed consent to the study, persons with dementia must assent to participation, as shown by their willingness to have the oils applied.

### Setting

The study will be conducted in mainstream and psychogeriatric ACFs in south-east Melbourne. Preference will be given to larger facilities (≥60 beds).

### Recruitment

Aged care facilities will be contacted by telephone to explain the study. If ACFs show interest in the study, researchers will visit the facility to provide all relevant parties (Director of Nursing, nursing staff and diversional therapists) with more detailed written and oral information. If facilities agree to participate in the study then approval is sought from the relevant ethics committee. Following approval, a pre-selection screening is conducted to identify eligible participants. An appointed delegate of the ACF will then contact the person responsible (PR) for each eligible person to ask if they consent with the forwarding of their contact details to a Monash University researcher. When verbal consent is given, a senior researcher will contact the PR to explain the study and answer queries. If a PR expresses interest in the study they are sent a Participant Information and Consent Form package, that includes detailed information on the study procedure, a consent form and pre-paid addressed return envelope. Once written consent is received from the PR, researchers gather baseline information to confirm eligibility of the person before enrolment in the study.

### Participants

Evidence suggests that verbally disruptive behaviours (e.g. calling out) link more with lowered mood while physically disruptive behaviours (e.g. pacing) link more to a lack of meaningful occupation and socialisation [18]. While these types of behaviours often occur together, there is a trend to distinguish between them for research purposes. The current study focuses on physically disruptive behaviours, because they are more common in ACFs. Selected participants must therefore display at least one high frequency, physically agitated behaviour that occurs daily at times other than during nursing interventions, and to a degree that requires staff intervention. Up to two target behaviours will be selected per participant in discussion with nursing staff based on frequency and severity using the Cohen Mansfield Agitation Inventory [19].

#### Inclusion criteria

(1) A chart diagnosis of moderate to severe dementia.

(2) Standard cognitive tests are invalid in this group since most participants will be severely impaired. Many will score zero on the widely used Mini-Mental State Examination [20]. The chart diagnosis of dementia will additionally be confirmed by interviewing staff with the Clinical Dementia Rating [21].

(3) At least one high frequency behavioural symptom as described above.

(4) An assessment by the ACF staff, GP and/or psychiatrist that behaviours are not due primarily to untreated or inadequately treated pain, physical illness, major depression or psychosis.

(5) Residence in a high care, or mixed high care and low care ACF for three or more months.

(6) Consent to the study by the PR as defined by the Victorian Civil and Administrative Tribunal.

#### Exclusion criteria

(1) Active treatment that might change over the study period by a psychiatrist or aged mental health team.

(2) A current, acutely life-threatening physical illness as reported by ACF staff and the GP.

(3) Behaviours that present a hazard to researchers (e.g. unpredictable aggression).

(4) A medical condition (e.g. dermatitis) that precludes the use of topical oil.

### Interventions

Consented, willing participants will be assigned in random order to one block of 30% lavender oil and one block of neutral control oil. Each block will last for three days (i.e. 3 observation/treatment periods) over a week to capture possible cumulative benefits, with at least a two-day washout period between each block. Thus, in total there will be 6 observations/treatment periods over a 2 week period. Interventions will be delivered at times when nursing staff report that the target behaviour/s are of high frequency (excluding times of personal nursing care).

A nurse will massage 1.0 ml of the 30% lavender or control oil into the resident's forearms for one minute. Based on an earlier study, we expect that lavender plasma levels will peak 20 minutes later and have faded by 90 minutes [7]. Observations will commence 30 minutes pre-exposure and finish 60 minutes post treatment, giving a total observation period of 90 minutes per session. A longer period is not feasible due to interruptions for nursing care, meals, visitors etc.

#### Materials

Aromatherapy will fail if the quantities of essential oil are too small to be effective. When applied by massage, lavender is typically prescribed as a 2.5% oil or lotion but there is no evidence to support this choice. We will apply 1 ml of 30% preparation to reduce the risk of missing a true effect through under-dosing. Pure lavender oil will be supplied by Essential Therapeutics, Melbourne, whose chemist will verify its purity using gas chromatography and supply a report to this effect. The characteristic profile of pure lavender's constituents is readily available [7].

Many varieties of pure lavender are held in stock. To select the one with greatest biological activity, up to 6 varieties will be assayed by Prof. George Lees (Department of Pharmacology and Toxicology, Otago University) who will measure the changes induced by 1:1000 dilutions of oils on the synaptic currents and action potentials of cultured networks of rat embryo pyramidal cortical neurones. The assays measure the capacity of samples to exert depressant effects on neurones, thus providing an animal model of sedation. The same assays have been used to demonstrate the mode of action of licensed antidepressants [22] and sedatives [23,24].

The selected pure lavender oil will then be diluted with jojoba oil. The control oil will comprise just the base ingredients and will be tested to ensure that it is biologically inert. The supplied products will be stored in opaque containers at the recommended temperature.

### Outcomes measures

A discretely positioned, trained researcher will record if the selected physically agitated behaviour is present or absent at one-minute intervals over the 90-minute observation period. Behaviour counts will range from zero to 30 in the *before *intervention period and zero to 60 in the *after *intervention period. This method was used successfully in previous studies with high levels of inter-rater reliability [2,25-28].

The *primary *measure in this trial will be the change in mean counts of physically agitated behaviours before and after intervention phases.

*Secondary *measures will include:

(a) A rating scale completed by the observing researcher during the 90 minute observation period at one-minute intervals of the participant's predominant type of affect. We will include three positive emotions (pleasure, contentment and interest), one neutral and three negative emotions (anger, sadness and fear/anxiety) [29].

(b) The 29-item Cohen-Mansfield Agitation Inventory [19] completed by the researcher in discussion with nursing staff in closest contact with the resident at the end of each week, relating to behaviour in the preceding week. Where possible, the same staff members will be questioned on each occasion.

(c) The proportion of residential facilities that continue to administer lavender as a treatment for agitation after the end of the trial.

(d) The cost of materials.

### Blinding and reliability

The order of conditions will be generated randomly using Random Number selection in Excel. An independent researcher will prepare the oil vials and no member of the lavender research team will have access to the code until the trial is completed.

The oils will be identical in appearance and texture, but nursing staff and possibly even the observers may detect by smell which treatment condition has been applied. To exclude this possibility, both the individual applying the oil and the observer will (at regular intervals) apply a cream containing a mixture of lavender and other essential oils (e.g. rosemary) to their upper lip while measuring participants' behaviour. In addition, nursing staff will wear a nose clip during the few minutes it takes to apply the oil to the resident's forearms.

Behaviour counts require some judgement and observers must be trained and monitored. To ensure high reliability (*kappa *> .90), a second research assistant will co-rate behaviours in the initial sessions following training by an experienced observer. Subsequent debriefing sessions and repeated co-rating will determine how much joint training and supervision is required to achieve this level of agreement.

### Sample size calculation

Sample size was calculated for the primary outcome measure (physically agitated behaviour) for a two-sided hypothesis test with a Type I error rate of 0.05 and a Type II error rate of 0.10 (90% power). In the following calculations, we used estimates for this outcome measure from a repeated measures study we conducted of family simulated presence therapy in a similar population [25]. In that study, the largest effect was achieved with simulated presence (effect size 0.45 relative to an attention control condition) with individualised music achieving a smaller effect size (effect size 0.32 relative to control). Given the mixed evidence supporting lavender oil, it seems prudent to apply the smaller effect of individualised music in the current study.

In the 30-minute baseline observation period, we anticipate that participants will display a mean count of 5.0 physically agitated behaviours per observation period with a standard deviation of 2.15. We anticipate a mean improvement of 0.69 behaviours per period during the control intervention compared with a mean improvement of 1.23 per period with lavender, giving a difference of 0.54 between the conditions (effect size 0.32). Based on data from our previous study, we estimate a within-person correlation of 0.70. A sample of 77 participants will be required to detect this difference, assuming the same variance (SD = 2.15) across conditions. Based on earlier experience and the brief duration of this study we expect 10% attrition and so will recruit 85 participants.

### Statistical analysis

We will use two-way repeated measures analysis of variance to test the significance of changes in the number of physically agitated behaviours over time (before and after treatment). We will use simple main effects to determine the specific effect of lavender relative to the control condition and we will test for the interaction between treatment and time. Simple contrasts will be used to tease out more detailed relationships, including testing the primary hypothesis that lavender oil reduces the frequency of physically agitated behaviours significantly more than a plausible control. Baseline characteristics of participants who drop out during the study will be compared to those who complete it to assess patterns of loss to follow-up and provide insights into the degree to which results can be generalised.

## Discussion

The proposed study will help meet the need for better controlled trials of alternative treatments for agitated behaviours associated with dementia. Our findings may guide family and professional carers in their selection of available evidence-based ways to reduce stressful behavioural symptoms that respond only partially to psychotropic medications. If our results show that use of Lavender oil is effective in reducing challenging behaviours in individuals with dementia, it will potentially provide a safer intervention rather than reliance on pharmacology alone. The study's findings will translate easily to other countries and cultures.

## Competing interests

The authors declare that they have no competing interests.

## Authors' contributions

DOC designed the original study. BE is project manager of the study, assisted by EvdP. All authors contributed to the writing of this paper and have approved the final version.

## Pre-publication history

The pre-publication history for this paper can be accessed here:

http://www.biomedcentral.com/1471-2318/10/49/prepub
